# Ubiquitylation of unphosphorylated c-myc by novel E3 ligase SCF^Fbxl8^

**DOI:** 10.1080/15384047.2022.2061279

**Published:** 2022-04-19

**Authors:** Sagar Bajpai, Hong Ri Jin, Bartosz Mucha, J. Alan Diehl

**Affiliations:** a Department of Biochemistry and Molecular Biology, Hollings Cancer Center, Medical University of South Carolina, Charleston, SC, USA; b Department of Biochemistry and Case Comprehensive Cancer Center, Case Western Reserve University, Cleveland, OH, USA

**Keywords:** Fbxl8, c-myc, ubiquitin ligase, cell cycle, cancer

## Abstract

Overexpression of c-myc via increased transcription or decreased protein degradation is common to many cancer etiologies. c-myc protein degradation is mediated by ubiquitin-dependent degradation, and this ubiquitylation is regulated by several E3 ligases. The primary regulator is Fbxw7, which binds to a phospho-degron within c-myc. Here, we identify a new E3 ligase for c-myc, Fbxl8 (F-box and Leucine Rich Repeat Protein 8), as an adaptor component of the SCF (Skp1-Cullin1-F-box protein) ubiquitin ligase complex, for selective c-myc degradation. SCF^Fbxl8^ binds and ubiquitylates c-myc, independent of phosphorylation, revealing that it regulates a pool of c-myc distinct from SCF^Fbxw7^. Loss of Fbxl8 increases c-myc protein levels, protein stability, and cell division, while overexpression of Fbxl8 reduces c-myc protein levels. Concurrent loss of Fbxl8 and Fbxw7 triggers a robust increase in c-myc protein levels consistent with targeting distinct pools of c-myc. This work highlights new mechanisms regulating c-myc degradation.

## Introduction

The c-myc protein is dysregulated in about 70% of all human cancers and is often considered a driver of human cancers. This stems from the fact that c-myc oncoprotein occupies 15% of the total genome promoters and plays a heavy role in many contexts of tumorigenesis including cell cycle, differentiation, proliferation, and metabolism.^1^ The significance of c-myc deregulation is documented in T cell acute lymphoblastic leukemia, multiple myeloma, and certain subsets of Burkitt’s lymphoma.^2^ Its involvement in such aggressive malignancies suggests that therapeutic efforts aimed at inhibiting MYC expression or activity should have an important clinical relevance. However, attempts to directly disrupt MYC function have been met with limited pharmacological success as there are still no clinically available MYC targeting therapies.^3^

C-myc is a highly labile protein with a half-life of less that 30 minutes.^4^ Degradation of c-myc occurs via the ubiquitin-proteasome system. Rapid protein turnover by the UPS is an essential mechanism responsible for tight control of physiological levels of c-myc.^5^ A well-defined process in c-myc degradation is the sequential phosphorylations of the serine 62 (S62) and threonine 58 (T58) residues. c-myc is stabilized upon phosphorylation of S62 by ERK.^6,7^ This priming step by ERK allows for the subsequent phosphorylation at the T58 residue by GSK3β.^6^ The S62 residue is then dephosphorylated by PP2A. This monophosphorylated c-myc (T58 only) is recognized by the E3 ubiquitin ligase Fbxw7 and degraded by the 26S proteasome.^8,9^ Highlighting the importance of this degradation pathway in cancer, many of the signaling proteins implicated in the MYC S62/T58 phosphorylation are often deregulated in tumor cells, resulting in altered c-myc phosphorylation and increased c-myc protein stability.^5,7^

In this study, we describe the identification and characterization of Fbxl8, an F-box protein that has only just recently been shown to function as an E3 ubiquitin ligase. As described below, Fbxl8 directly polyubiquitylates c-myc independent of the canonical phosphorylation sites (T58 and S62) in vitro and in vivo. Loss of Fbxl8 results in c-myc accumulation and cell cycle dysregulation, revealing tumor suppressor potential.

## Results

### c-Myc associates with Fbxl8 in vivo

To identify Fbxl8 substrate targets, Flag-tagged Fbxl8 was expressed in 293 T cells; Flag-Fbxl8 was precipitated from lysates, and coprecipitating proteins were identified by mass spectroscopy analysis to identify bound protein partners. Among numerous coprecipitating proteins, the presence of the oncogenic transcription factor, c-myc, was of particular interest (Supplemental Figure 1). C-myc stability and activity are regulated by numerous E3 ligases, and the possibility that Fbxl8 might contribute to the regulation of c-myc warranted further investigation.

**Figure 1.**
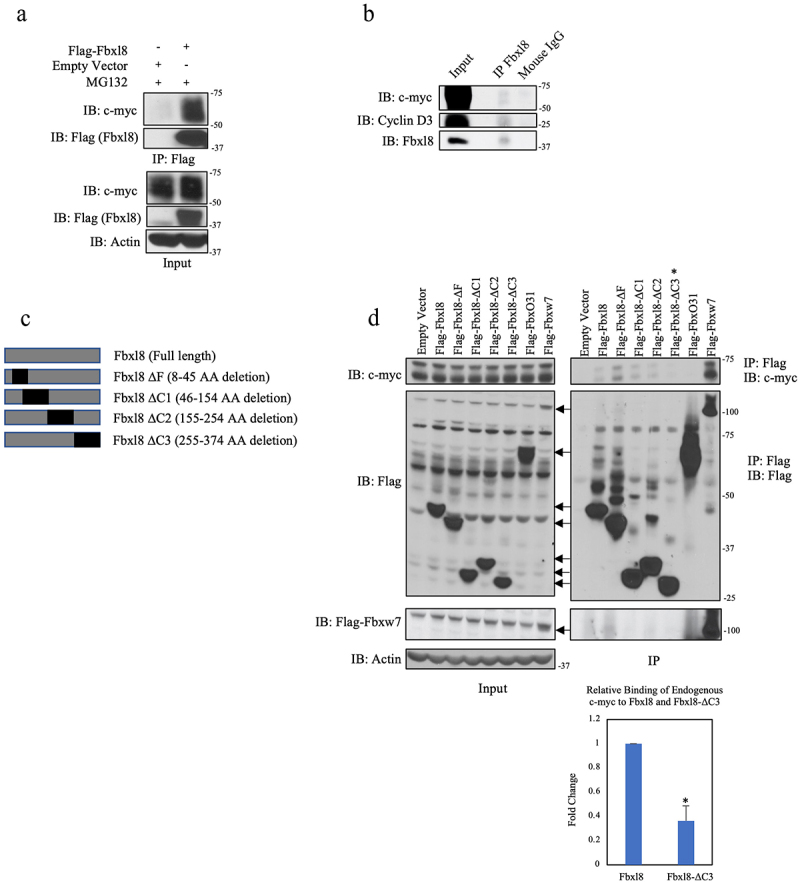
**C-myc associates with Fbxl8**. (a) Lysates from U2OS cells transfected with an empty vector or Flag-Fbxl8 and treated with MG132 (10 μM) for 6 hours were immunoprecipitated with anti-Flag antibodies. Immune complexes were analyzed by western blot. (b) Lysates from U2OS cells were immunoprecipitated with anti-Fbxl8 antibody crosslinked to Protein A Sepharose beads. Immune complexes were analyzed for c-myc and Cyclin D3 by western blot. (c) Schematic model of deletion mutants of Flag-tagged Fbxl8 construct. (d) Lysates from U2OS cells transfected with an empty vector, Flag-Fbxl8, and deletion mutants and treated with a proteasome inhibitor MG132 (10 μM) for 6 hours were immunoprecipitated with anti-Flag beads. Immune complexes were analyzed by western blot. Densitometric quantification of binding between endogenous c-myc and ectopic Fbxl8-ΔC3 relative to ectopic wild-type Fbxl8 control (Ratio Paired t-test, p-value < 0.05; N = 3).

We first confirmed c-myc-Fbxl8 binding following coexpression in U2OS cells (Figure 1(a)). We subsequently assessed binding of endogenous c-myc and Fbxl8. Endogenous Fbxl8 was precipitated from U2OS cells, and the presence of c-myc was assessed by immunoblot. As a positive control, we assessed coprecipitation of cyclin D3 since we have already established cyclin D3 as a direct Fbxl8 substrate.^10^ Both c-myc and cyclin D3 were detected in Fbxl8 precipitates (Figure 1(b)). To address binding specificity, we assessed the ability of c-myc to bind to a panel of Fbxl8 deletion mutants. Specific mutations of interest include c-terminal deletions that should impact Fbxl8 binding with substrates (ΔC1-3; Figure 1(c)). Flag-tagged wild-type and mutant Fbxl8 expressing vectors were expressed in U2OS cells; we also expressed Fbxw7 as a positive control versus Fbxo31 as a negative control. Flag-Fbxl8-ΔF, a catalytically deficient mutant lacking its F-box motif and therefore unable to bind with Skp1, was also overexpressed. Cells were treated with MG132 to inhibit degradation of c-myc prior to generating cell lysates. Complexes were precipitated with Flag antibodies, and coprecipitating, endogenous c-myc was assessed by immunoblot (Figure 1(d)). Binding of c-myc with Fbxw7 and wild-type Fbxl8 was readily detected with no binding of c-myc with Fbxo31 (Figure 1(c)). A significant loss of c-myc-Fbxl8 association was observed, with the Fbxl8-ΔC3 mutation demonstrating a c-myc binding domain within residues 255–374 of Fbxl8 (Figure 1(d)).

### SCF^Fbxl8^ polyubiquitylates c-Myc

**Figure 2.**
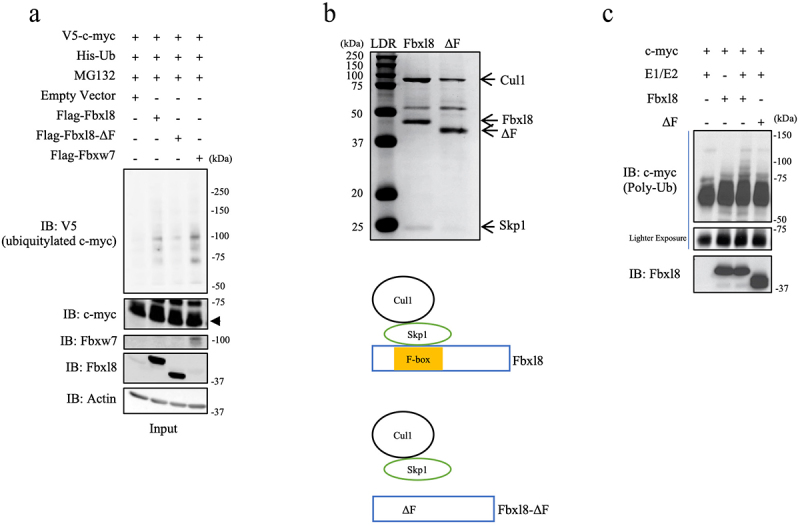
**C-myc is a SCF^Fbxl8^ substrate**. (a) Lysates from U2OS cells cotransfected with His-Ub, pBABE-c-myc, and Fbxl8, Fbxl8-ΔF, Fbxw7, or Empty Vector were treated with MG132 (20 μM) for 6 hours prior to immunoprecipitation with anti-His beads. Immune complexes were analyzed by western blot for ubiquitinated c-myc (anti c-myc) and F-box proteins (anti-Flag), and β-actin was a loading control. (b) Coomassie stain verifying purified SCF (Skp1-Cul1-F-box) complexes from SF9 cells for Fbxl8 and Fbxl8-ΔF. (c) In vitro ubiquitylation assay was performed in reaction mixtures containing the presence or absence of indicated mixture components. Lysates from assays were analyzed by western blot using antibodies against indicated proteins.

F-box proteins frequently function as substrate specific adaptors of the Skp-Cullin1 E3 ligase. We hypothesized that Fbxl8 in association with the Skp-Cullin1 E3 ligase (SCF^Fbxl8^) might direct c-myc polyubiquitylation. To test this, wild-type Fbxl8, Fbxl8-ΔF (inactive mutant), and c-myc were overexpressed in U2OS cells along with His-tagged ubiquitin. Fbxw7 was used as a positive control for c-myc polyubiquitylation. Following transfection, cells were treated with MG132 and complexes were precipitated from cell lysates with antibodies directed to the His-tagged ubiquitin. We noted increased c-myc polyubiquitin chains in the presence of wild-type Fbxl8 and reduced polyubiquitin chains with Fbxl8-ΔF (Figure 2(a)). To determine if polyubiquitylation was direct, we purified SCF^Fbxl8^ or SCF^Fbxl8ΔF^ from Sf9 cells (Figure 2(b)). Recombinant, purified SCF^Fbxl8^ but not SCF^Fbxl8ΔF^ polyubiquitylated purified c-myc (Figure 2(c)), demonstrating that c-myc is a direct SCF^Fbxl8^ substrate.

### SCF^Fbxl8^ polyubiquitylates c-Myc independent of phosphorylation at threonine 58 and serine 62

Canonical c-myc polyubiquitylation is catalyzed in an Fbxw7-dependent fashion.^8^ Binding of Fbxw7 with c-myc is dependent upon phosphorylation of serine 62 (S62) and threonine 58 (T58) residues by Erk1/2 and GSK3β.^9^ c-myc is stabilized upon phosphorylation of S62 by ERK.^11,12^ However, phosphorylation of S62 serves as a priming event for the subsequent phosphorylation of the T58 residue by GSK3β^6^ after which the S62 residue is then dephosphorylated by PP2A.^7^ Threonine 58 phosphorylated c-myc is recognized by SCF^Fbxw7^, polyubiquitylated, and degraded by the 26S proteasome.^9,13^

**Figure 3.**
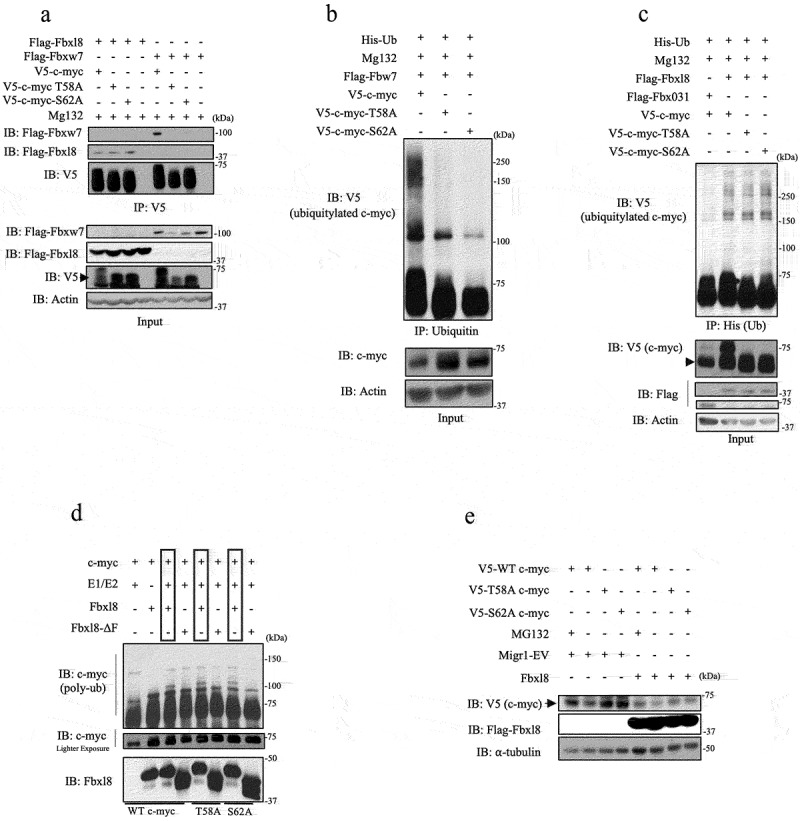
**SCF^Fbxl8^ polyubiquitylates c-myc independent of phosphorylation at threonine 58 and serine 62**. (a) Lysates from U2OS cells transfected with either Flag-Fbxl8 or Flag-Fbxw7 along with c-myc and c-myc phosphomutants and treated with MG132 (20 μM) for 6 hours were immunoprecipitated with anti-V5 beads. Immune complexes were analyzed by western blot. (b) Lysates from U2OS cells cotransfected with His-Ub, His-V5-c-myc WT, T58A or S62A, and Fbxw7 were treated with MG132 (20 μM) for 6 hours prior to immunoprecipitation of ubiquitin with TUBEs (Tandem Ubiquitin Binding Entities). Immune complexes were analyzed by western blot for ubiquitinated c-myc and c-myc phosphomutants (anti-V5) and β-actin as a loading control. (c) Lysates from U2OS cells cotransfected with His-Ub, His-V5-c-myc WT, T58A or S62A, and either Fbxl8 or Fbxo31 (negative control) were treated with MG132 (20 μM) for 6 hours prior to immunoprecipitation with anti-His beads. Immune complexes were analyzed by western blot for ubiquitinated c-myc and c-myc phosphomutants (anti-V5), F-box proteins (anti-Flag), and β-actin as a loading control. (d) In vitro ubiquitylation assay was performed in reaction mixtures containing the presence or absence of indicated mixture components. Lysates from assays were analyzed by western blot using antibodies against indicated proteins.

To evaluate the potential role of SCF^Fbxl8^ in c-myc metabolism, we first assessed whether Fbxl8-c-myc binding was regulated by T58/S62 phosphorylation. We compared binding of Fbxl8 with V5 tagged wild-type c-myc versus either c-myc-T58A or c-myc-S62A. We also evaluated binding of Fbxw7 with c-myc mutants as a control. Complexes were precipitated from U2OS cells expressing relevant expression plasmids. Complexes were collected by precipitation with antibody directed to the V5 tag followed by western blot analysis. As previously published, Fbxw7 binding to c-myc was abolished by mutation of either S62 or T58 (Figure 3(a)). Strikingly, Fbxl8 associated with c-myc independent of S62 or T58 (Figure 3(a)), suggesting that Fbxl8 might regulate c-myc in a phosphorylation-independent fashion.

To further evaluate the role of c-myc phosphorylation in the context of regulation by Fbxl8, we next determined the contribution of T58 and S62 for polyubiquitylation by SCF^Fbxl8^ both in vivo and in vitro. We evaluated c-myc versus c-mycT58A or c-mycS62A polyubiquitylation by either Fbxw7 or Fbxl8 in U2OS cells. While Fbxw7, as previous published, efficiently promoted wild-type c-myc polyubiquitylation and failed to trigger ubiquitylation of either c-mycT58A or c-mycS62A (Figure 3(b)), in contrast, Fbxl8 expression efficiently promoted polyubiquitylation of both wild-type and mutant c-myc (Figure 3(c)). Analogous with the in vivo analysis, purified SCF^Fbxl8^ polyubiquitylated c-myc independent of S62 and T58 (Figure 3(d)), demonstrating that SCF^Fbxl8^ catalyzes c-myc ubiquitylation independent of canonical phosphorylation sites. This observation was further complemented with Fbxl8 overexpression reducing protein levels of overexpressed c-myc-T58A and c-myc-S62A (Figure 3(e)).

### Fbxl8 regulates c-Myc accumulation in a 26S Proteasome-Dependent Manner

**Figure 4.**
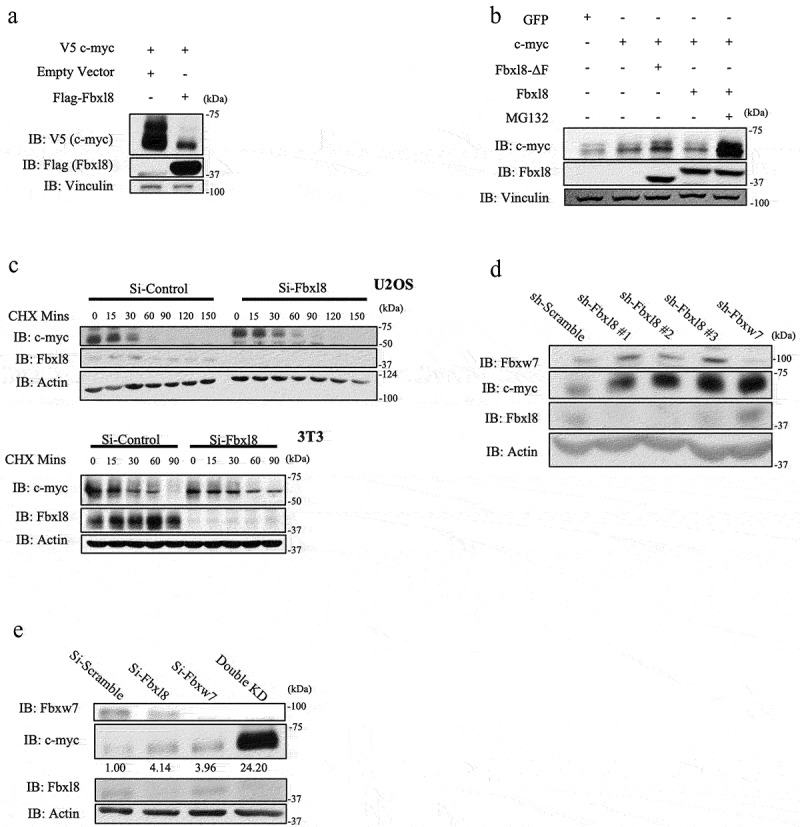
**Fbxl8 regulates c-Myc accumulation in a proteasome-dependent manner**. (a) Lysates from 293 T cells were transfected with either an empty vector or Fbxl8, along with wildtype c-myc. Exogenous c-myc levels were analyzed via western blot along with Fbxl8 and Vinculin. (b) Lysates from 293 T cells transfected with either GFP or c-myc along with Fbxl8 or Fbxl8-ΔF were analyzed via western blot. (c) Lysates from 3T3 and U2OS treated with either si-Control or si-Fbxl8 and cycloheximide 100 μg/mL over indicated time periods and analyzed via western blot for endogenous c-myc, Fbxl8, and Vinculin. (d) Lysates from U2OS cells infected with scramble shRNA, sh-Fbxl8, or sh-Fbxw7. Endogenous c-myc, Fbxl8, and β-actin were analyzed via western blot. (e) Lysates from U2OS cells treated with si-Scramble si-Fbxl8 or si-Fbxw7 for 3 days and analyzed by western blot for endogenous c-myc, Fbxl8, Fbxw7, and β-actin. C-myc levels were quantified via densitometry, and presented values are presented relative to si-Scramble control.

Because ubiquitylation frequently regulates protein degradation, we assessed the impact of Fbxl8 on c-myc accumulation. HEK293T cells were transfected with either V5-tagged c-myc, and Fbxl8 c-myc levels were followed by western blot. Expression Fbxl8 significantly reduced levels of c-myc (Figure 4(a)). We subsequently evaluated the impact of expression of wild-type Fbxl8 versus Fbxl8-ΔF on c-myc. Expression of wild-type Fbxl8 reduced c-myc levels in a 26S proteasome-dependent manner (Figure 4(b)). To determine if differential accumulation of c-myc reflects protein degradation, we measured c-myc half-life following Fbxl8 knockdown in both human U2OS cells and murine fibroblasts. As expected, Fbxl8 knockdown significantly extended the half-life of c-myc in both cell types (Figure 4(c)).

We next addressed the relative contribution of Fbxl8 and Fbxw7 to c-myc accumulation. Initially, we tested 3 independent shRNA’s targeting Fbxl8 relative to knockdown of Fbxw7 and noted similar accumulation of c-myc under all knockdown conditions (Figure 4(d)). Because Fbxw7 and Fbxl8 target different populations of c-myc (phosphorylated versus unphosphorylated), we hypothesized that concurrent knockdown of both would have an additive impact on c-myc accumulation. Indeed, knockdown of both Fbxl8 and Fbxw7 significantly increased c-myc levels above that observed with knockdown of either individual F-box protein (Figure 4(e)).

### Fbxl8 knockout elevates c-Myc and accelerates G1/S Phase Transition

**Figure 5.**
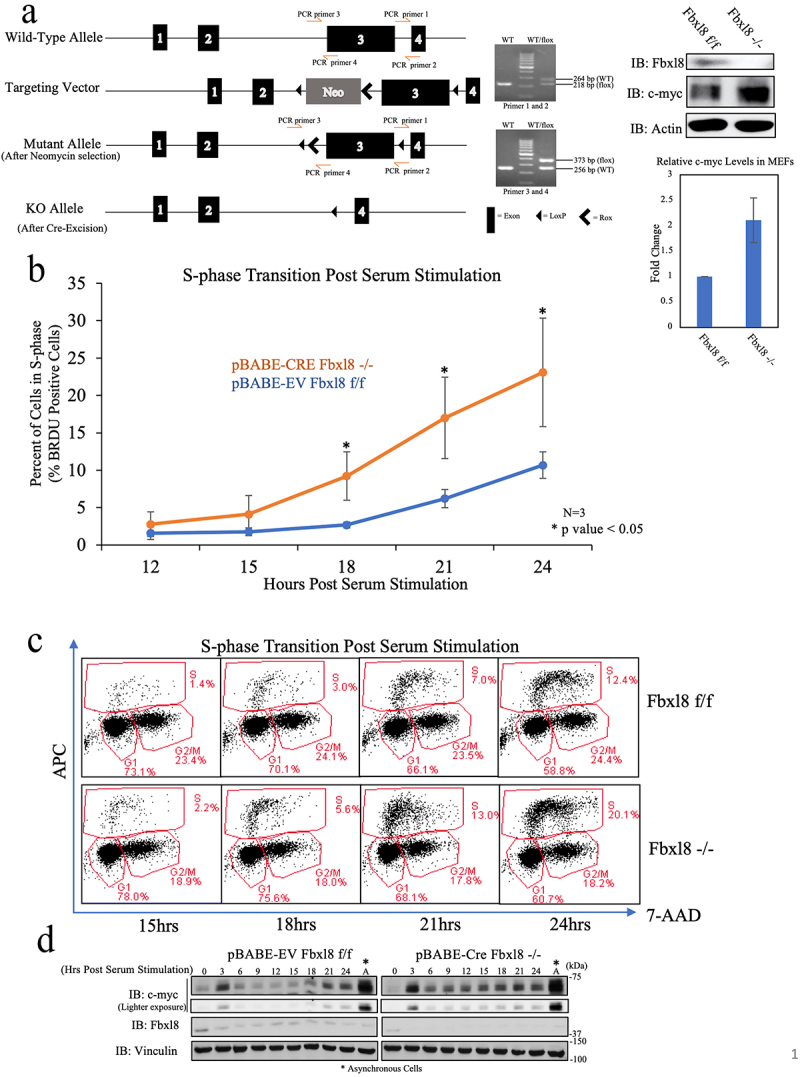
**Fbxl8 excision accelerates G1/S phase transition**. (a) Schematic for generation of conditional knockout Fbxl8 allele. Lysates from Fbxl8^f/f^ and Fbxl8^−/−^ MEFs were analyzed by western blot to determine Fbxl8 and c-myc protein levels after Fbxl8 ablation from the genome. Densitometric quantification of c-myc in Fbxl8^f/f^ and Fbxl8^−/−^ MEFs (Ratio Paired t-test, p-value < 0.05; N = 3). (b) Fbxl8^f/f^ and Fbxl8^−/−^ murine embryonic fibroblasts arrested at G0/G1 by contact inhibition and serum starvation for 36 hours. S-phase entry was assessed by BRDU incorporation (30 min) and FACS at 12, 15, 18, 21, and postrelease. Quantification of BRDU-positive cells mean ± SD, *p < .05 (two-tailed Student’s *t*  test, *n* = 3). (c) Flow cytometry data of specified timepoints postserum stimulation. (d) Western analysis of lysates from (B) for c-myc, Fbxl8, and vinculin.

C-myc is an immediate early gene, and c-myc accumulation is necessary for cell cycle progression. In cells with activated c-myc, cell division accelerates primarily during the G1 to S-phase interval.^14^ Fbxl8 knockdown also accelerates G1 to S-phase entry in mammalian cells.^10^ Therefore, it was critical to investigate the status of c-myc protein levels in this cell cycle-specific context. We obtained conditional knockout mice generated by Cyagen, wherein exon 3 (which encodes the F-box domain) of the Fbxl8 gene is flanked by LoxP sequences (Figure 5(a)). A detailed description of the phenotype of Fbxl8 knockout mice will be provided subsequently. For the current work, we generated murine embryonic fibroblasts (MEFs) from mouse embryos homozygous for floxed Fbxl8 alleles (Fbxl8^f/f^). MEFs were infected with control retrovirus or retrovirus encoding Cre recombinase after which we assess levels of Fbxl8 and c-myc. Expression of Cre reduced Fbxl8 levels below detection levels and triggered elevation of c-myc protein levels. (Figure 5(a)). To address the impact of Fbxl8 knockout on cell cycle progression, Fbxl8^f/f^ and Fbxl8^−/−^ MEFs were arrested in G0/G1 via serum starvation and contact inhibition. Cell cycle reentry was analyzed at 0–24 hours following mitogenic stimulation (Figure 5(b-c)). As expected, Fbxl8^−/−^ MEFs exhibited an accelerated G1 to S phase transition along with higher c-myc protein levels (Figure 5(d)). Inhibition of c-myc in Fbxl8^−/−^ cells vastly reduced G1/S phase transition (Supplemental Figure 3C), substantiating evidence that Fbxl8ʹs activity on the cell cycle is additionally mediated through c-myc protein levels.

### Reduced Fbxl8 levels correlate with poor survival in cancer patients

**Figure 6.**
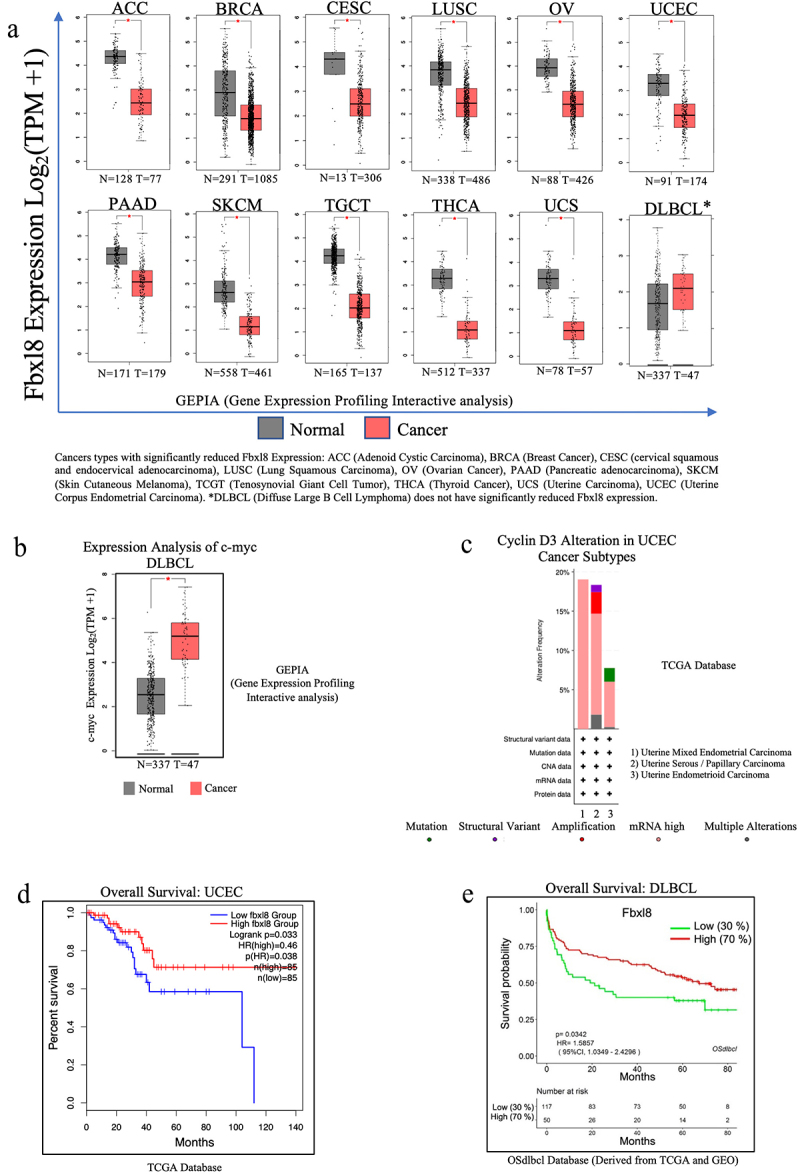
**Fbxl8 levels correlate with poor survival of DLBCL and UCEC patients**. (a) Fbxl8 expression analysis for various cancer types comparing normal versus tumor tissue in patients. Cancer types include ACC (Adenoid Cystic Carcinoma), BRCA (Breast Cancer), CESC (cervical squamous and endocervical adenocarcinoma), LUSC (Lung Squamous Carcinoma), OV (Ovarian Cancer), PAAD (Pancreatic adenocarcinoma), SKCM (Skin Cutaneous Melanoma), TCGT (Tenosynovial Giant Cell Tumor), THCA (Thyroid Cancer), UCS (Uterine Carcinoma), UCEC (Uterine Corpus Endometrial Carcinoma), and DLBCL (Diffuse Large B-cell Lymphoma). (b) c-myc expression analysis for DLBCL comparing normal versus tumor tissue in patients. (c) Alteration frequency of cyclin D3 in uterine corpus endometrial carcinoma (UCEC). (d) Kaplan–Meier survival curve for overall survival in diffuse large B-cell lymphoma (DLBCL) patients with low and high expression of Fbxl8. (e) Kaplan–Meier survival curve for overall survival in uterine corpus endometrial carcinoma patients with low and high expression of Fbxl8.

Given that Fbxl8 negatively regulates two oncogenes, cyclin D3 and c-myc, we hypothesized that Fbxl8 levels might be reduced in certain cancers.^10^ We therefore assessed Fbxl8 levels in the TCGA data base. While we did not observe a high frequency of recurrent mutations in Fbxl8, we noted that expression of Fbxl8 was significantly reduced in a number of cancers (Figure 6(a)). C-myc is frequently overexpressed in Diffuse Large B-cell Lymphoma (DLBCL) (Figure 6(b)), while cyclin D3 is often mutated or overexpressed in Uterine Corpus Endometrial Carcinoma (UCEC) subtypes (Figure 6(c). We therefore assessed patient survival versus Fbxl8 levels in these two cancer types. Indeed, reduced Fbxl8 levels correlated with poor patient prognosis (Figure 6(d, e)). While correlative, this is consistent with the role of Fbxl8 as an antagonist of key tumor drivers and suggests that its loss may in fact contribute to tumor progression.

## Discussion

C-myc is a potent oncoprotein, overexpression of wild-type c-myc triggers cancer in model systems, and this occurs in many human cancers where it is considered a driver oncogene.^15,16^ Given the predominant protumor function of c-myc, it is not surprising that c-myc functions as an integrative convergence for extracellular stimuli and as a master regulator of many downstream cellular responses and pathways. Regulation of c-myc occurs at multiple levels including transcription, translation, and protein degradation.^17^ The c-myc protein is highly labile, and a common feature of labile regulators of cell growth and survival is ubiquitin-dependent post-translational regulation. However, c-myc ubiquitylation can be catalyzed by several E3 ligases including Skp2, HUWE1, and Fbxw7 where Fbxw7 is the primary regulator of c-myc ubiquitin-dependent degradation.^18–20^ In addition, c-myc degradation is most closely associated with phosphorylation of key residues; phosphorylation of such residues generates a phospho-degron recognized by downstream E3 ligases. However, this begs the question of whether and how is unphosphorylated c-myc degraded. We have found that Fbxl8 functions to recognize unmodified c-myc and thereby regulate underphosphorylated pools of c-myc. Fbxl8 binds directly to unphosphorylated c-myc and coordinates polyubiquitylation of unphosphorylated c-myc in vitro and in vivo. Concurrent loss of Fbxl8 and Fbxw7 has an additive impact on c-myc levels consistent with them targeting the distinct population of c-myc for degradation. Our results suggest that Fbxw7 and Fbxl8 work in concert to maintain homeostatic c-myc levels. Finally, loss of Fbxl8 promotes c-myc overexpression and accelerates G1 phase progression. When c-myc is knocked down in Fbxl8^−/−^ cells, there is a significant reduction in cell cycle reentry. This makes c-myc the second known substrate target of Fbxl8 to regulate G1/S phase transition.

Canonical c-myc degradation is dictated by ERK and GSK3ꞵ kinases that sequentially phosphorylate c-myc at T58 and S62 residues. Mutation of either residue inhibits Fbxw7 binding and stabilizes c-myc, resulting in a dramatic increase in transcription of downstream target genes. This increase in pro-proliferative c-myc targets is clearly a driver of cancer as genetic inactivation of c-myc or use of Brd4 inhibitors, which also reduce c-myc levels, has significant antitumor activities.^21,22^

While c-myc is clearly a tumor promoter, too much uncontrolled c-myc can be toxic. There are a variety of potential c-myc activities that when unchecked can trigger cell death including disruption of DNA replication origin priming DNA damage and proteotoxicity.^23–25^ Proteotoxicity is reflected in c-myc’s function to regulate ribosome levels and overall rates of protein synthesis via its activity in polymerase I transcription and recruitment of polymerase II.^26,27^ Elegant work has revealed that protein translation checkpoints that limit protein translation rates are essential for the survival of c-myc overexpressing tumor cells.^28^ This highlights the importance of maintaining a critical threshold of c-myc protein in both normal and tumor cells for overall survival. Our data demonstrating regulation of unphosphorylated c-myc and phospho-c-myc by distinct E3 ligase pathways highlight new depth and importance to the context of c-myc turnover. The role of Fbxl8 in tumors with inactive Fbxw7 requires further investigation to ascertain the relative importance of the SCF^Fbxl8^ ligase in this context and whether there are therapeutic opportunities.

## Materials and methods

### Cell culture

NIH3T3 and 293 T were cultured in Dulbecco’s modified Eagle’s Medium (DMEM) supplemented with 10% fetal bovine serum (FBS), 100 units of penicillin, and 100 mg/ml of streptomycin (Gemini). U2OS cells were in McCoy’s 5A Medium supplemented with 10% fetal bovine serum (FBS), 100 units of penicillin, and 100 mg/ml of streptomycin (Gemini).

### Generation of Fbxl8 knockout allele

Murine embryonic fibroblasts (MEFs) were generated from conditional knockout mice provided by Cyagen. The Fbxl8 allele, specifically exon 3 (which encodes the F-box domain) in these mice, is flanked with LoxP sites sensitive to Cre-Recombinase activity. Mouse embryos from a pregnant Fbxl8^f/f^ mother were harvested at day 14, and cells were passed every 3 days until immortalization as dictated by the 3T9 protocol. Immortalized Fbxl8^f/f^ MEFs were then infected with pBABE-Cre recombinase viral particles, and excision of Fbxl8 alleles was analyzed via western blot.

### Vectors/plasmids

Full-length Flag-Fbxl8 and Fbxl8-mutant constructs, which were previously used by Yoshida et al. (2021), were cloned into pHAGE vector using a QuickChange site-directed mutagenesis kit (Stratagene, La Jolla, CA) using pcDNA3-Fbxl8 plasmid as a template. PCR reactions were performed following the manufacturer's instructions. Clones were sequenced in their entirety to confirm the presence of mutations. All the following c-myc constructs overexpressed in a pD40 vector were obtained from Addgene: pD40His-/V5-c-Myc WT (#45597), pD40-His/V5-c-MycT58A (#45598), and pD40-His/V5-c-MycS62A (#45599). Control Flag Fbxo31 and Flag Fbxw7 constructs in pcDNA vector were obtained from Michele Pagano Lab. For some experiments, His tag was deleted from pD40-His/V5-c-Myc-WT and pD40-His/V5-c-myc-T58A/S62 using site-directed mutagenesis protocol and reagents by GM Biosciences (GM7002) with the following primers:

5’-GATTCTACGCGTACCGGTTGAGTTTAAACCCGCTGA-3’

5’-TCAGCGGGTTTAAACTCAACCGGTACGCGTAGAATC-3’

SF9 viral particles for Fbxl8 and Fbxl8-ΔF constructs were generated from pHAGE-Flag-Fbxl8 and pHAGE-Flag-Fbxl8-ΔF constructs being cloned into PfastBac1 vector and then recombined with the Bacmid Cassette using the protocol and reagents from Bac-to-Bac Baculovirus Expression System (Thermofisher Cat# 10359016).

### RNAi

siRNA for scramble control (ON-TARGETplus Non-Targeting siRNA #1), Fbxl8 (SMARTpool: siGENOME Fbxl8 siRNA), and Fbxw7 (SMARTpool: siGENOME Fbxw7 siRNA) were purchased from Horizon. The shRNA for control (pLKO.1) was purchased from (addgene plasmid #10878). Fbxl8 shRNA (GIPZ Lentiviral Human Fbxl8 shRNA) and Fbxw7 shRNA (GIPZ Lentiviral Human FBXW7 shRNA) were also purchased from Horizon.

### Transfection, infection, and cell cycle analysis

For transfection, expression plasmids were transfected into cells with lipofectamine PolyJet (SigmaGen Laboratories Cat# SL100688) reagent according to the manufacturer’s instructions. For viral production, viral expression plasmids were cotransfected into 293 T cells with lipofectamine (ThermoFisher Scientific Cat# 18324012). Virus supernatants were harvested 48 to 72 hours after transfection and used to infect cells with polybrene (10 μg/μl). For cell cycle experiments, NIH 3T3 cells were synchronized in the G0/G1 phase by contact inhibition or DMEM containing 0.1% FBS for 36 hours at high density. The cell cycle was released from the G0/G1 phase by splitting cells with DMEM containing 10% FBS. Cells were harvested, fixed, and stained using Cytofix and permeabilization plus reagents from the APC BRDU Flow Kit (BD Pharmingen Cat# 552598). For BRDU detection, cells were cultured in medium containing 10uM BRDU for 30 minutes, fixed with Cytofix and Permeabilization Plus reagents (BD Pharmingen), and stained with an anti-BRDU monoclonal antibody conjugated with APC and 7-AAD for DNA staining. Cell cycle distribution and BRDU positive Cells were measured with a BD Accuri C6 Plus Flow Cytometer.

### Western analysis

Cells were lysed in EBC buffer (50 mM Tris pH 8.0, 120 mM NaCl, 1 mM EDTA, 0.5% NP40) containing 1 mM phenylmethylsulfonyl fluoride, 20 U/ml aprotinin, 1 µM leupeptin, 1 mM dithiothreitol, 0.1 mM NaF, 0.1 mM sodium orthovanadate, and 10 mM β-glycerophosphate. Proteins were resolved by SDS-PAGE, transferred to the membrane, and immunoblotted with the indicated antibodies: Fbxl8 (Santa Cruz Cat# sc-390582), Fbxw7 (Bethyl Laboratories Cat# A301-720A), c-myc (Cell Signaling Cat# 9402S), Cyclin D3 (Cell Signaling DCS22), Actin (Sigma Cat# A5316), vinculin (Cell Signaling Cat# 4650S), Alpha Tubulin (Sell Signaling Cat# 2144S) Anti-Flag (Sigma Cat#F7425-.2 MG), and anti-V5 (Sigma Cat#V8137-.2 MG). Proteins of interest were detected with horseradish peroxidase-conjugated antimouse or rabbit antibodies, and signals were visualized with the ECL system (Perkin Elmer Cat# NEL105001EA).

### Mass spectrometry analysis

293 T expressing Flag-Tagged Fbxl8 (wildtype) or Flag-Tagged Fbxl8-ΔF (mutant) cells were harvested and subsequently lysed in Tween 20 lysis buffer (50 mM HEPES (pH 8.0), 150 mM NaCl, 2.5 mM EGTA, and 1 mM EDTA, 0.1% Tween 20) with protease and phosphatase inhibitors (1 mM PMSF, 20 U/ml aprotinin, 5 mg/ml leupeptin, 1 mM DTT, 0.4 mM NaF, and 10 mM b-glycerophosphate). Fbxl8 complexes were purified using FLAG M2 Agarose (Sigma Cat No. A2220), separated by SDS Page, and stained with Pierce Silver Stain (Thermo scientific Cat# 24612). Gel pieces were cut and submitted to the Taplin Mass Spectroscopy Facility to determine peptide fragment sequences and hence protein identity of co-immunoprecipitated proteins. Spectral matches were manually examined and multiple peptides per protein were required.

### In-vitro ubiquitylation

SF9 (1 ✕ 10^8^) insect cells were infected with baculoviral Flag-Fbxl8 or Flag-ΔF along with remaining SCF components (Skp1, Cul1, and Rbx1) for 1 hour and harvested 40 hours post transfection. Cells were lysed in Tween 20 lysis buffer Harvested cells were lysed in Tween20 lysis buffer (50 mM HEPES (pH 8.0), 150 mM NaCl, 2.5 mM EGTA, 1 mM EDTA, 0.1% Tween 20) with inhibitors for protease and phosphatase (1 mM PMSF, 20 U/ml aprotinin, 5 mg/ml leupeptin, 1 mM DTT, 0.4 mM NaF, and 10 mM b-glycerophosphate) for 2 hours. Flag-Fbxl8 or Flag-ΔF was immunoprecipitated from sf9 lysates using anti Flag beads (Sigma Cat No. A2220). The beads were washed 5 times with Tween 20 lysis buffer and Flag-Fbxl8 or Flag-Fbxl8-ΔF was purified. Purified F-box proteins were incubated with c-myc substrate purified from 293 T cells for 2 hours at 37 degrees. Protein complexes were analyzed by western blot.

### Ubiquitination assay

For the in-vivo ubiquitination assay, U2OS Cells were transfected with indicated plasmids (Detailed plasmids are described in Figure legends) for 40 hours and treated with MG132 (10 μM) for 6 hours. Cell lysates in EBC buffer were subjected to immunoprecipitation with antibodies against His tagged ubiquitin followed by western analysis to detect the ubiquitinated protein targets.

### Co-Immunoprecipitations assays

U2OS cells were transfected with Flag-Fbxl8, Flag-Fbxl8-ΔF/ΔC mutants, Flag-Fbxw7, and Fbxo31 for 5 hours and harvested 40 hours post-transfection. Harvested cells were lysed in EBC buffer with inhibitors (1 mM PMSF, 20 U/ml aprotinin, 5 mg/ml leupeptin, 1 mM DTT, 0.4 mM NaF, and 10 mM b-glycerophosphate) for protease and phosphatase for 30 minutes. Flag-tagged F box proteins were precipitated from cell lysates using anti-Flag beads (Sigma Cat No. A2220). The beads were washed with EBC lysis buffer, and F-box proteins were purified. Endogenous binding was conducted by harvesting U2OS cells 5 hours after MG132 treatment. Fbxl8 was precipitated from lysates using Protein A/G beads (Pierce) crossing linked to Anti-Fbxl8 antibodies (Santa Cruz Cat# sc-390582) via disuccinimidyl suberate.

### Expression analysis and Kaplan–Meier overall survival data

Expression analysis was derived from TCGA and GTEx data and generated by the GEPIA (Gene Expression Profiling Interactive Analysis) webserver.^29^ Kaplan–Meier analysis wsas derived from the TCGA database for DLBCL and generated by the OSdlbcl webserver.^30^ Kaplan–Meier analysis for UCEC was derived from the TCGA database and generated by the UALCAN web platform.^31^ Alterations of Cyclin D3 in UCEC data were derived from TCGA database and generated by the cBio Cancer Genomics Portal platform.^32^

## Supplementary Material

Supplemental Material
