# Supplementary figures and images for: Ubiquitylation of unphosphorylated c-myc by novel E3 ligase SCFFbxl8
Source: Cancer Biol Ther. 2022 Apr 19;23(1):348–57. doi: 10.1080/15384047.2022.2061279 (PMC9037475; doi:10.1080/15384047.2022.2061279)

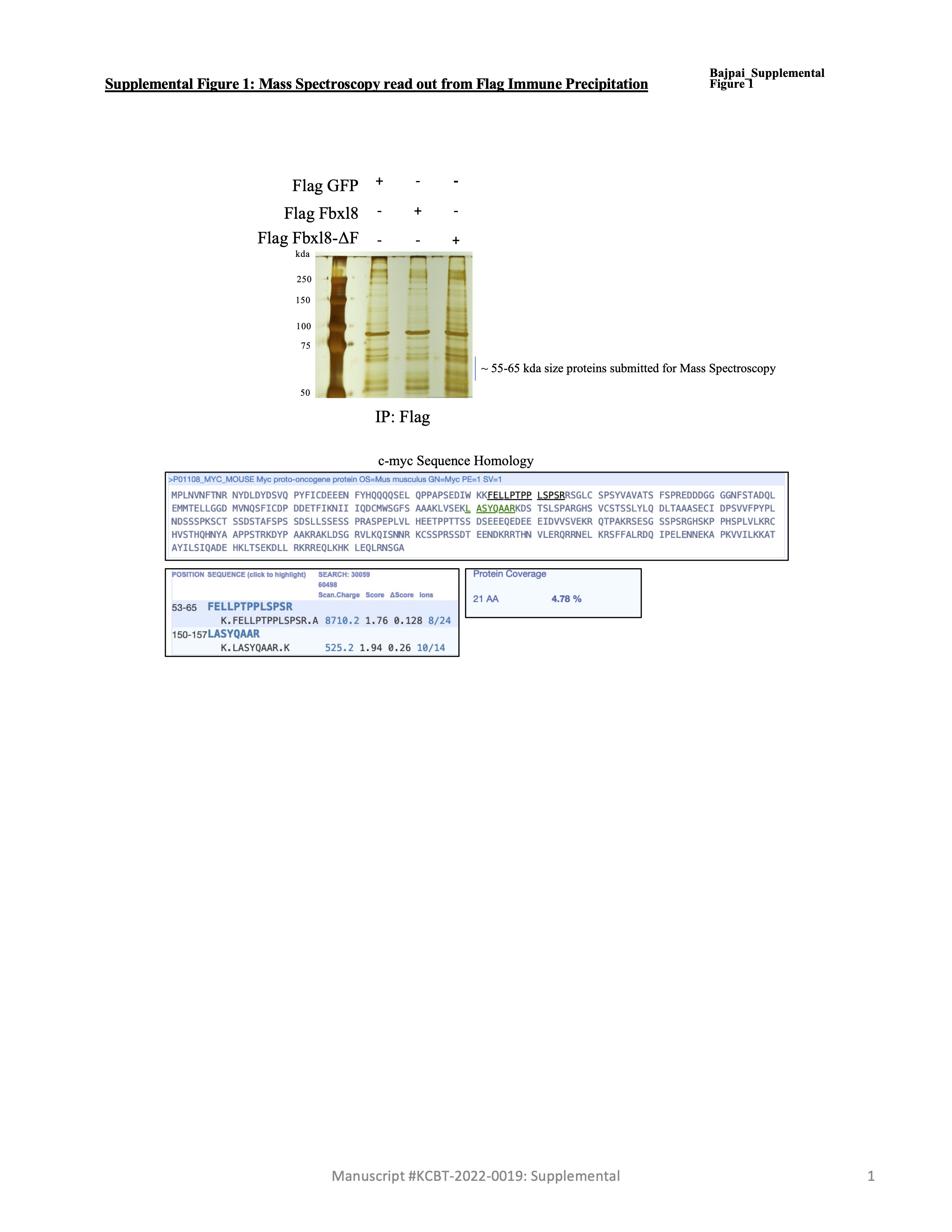

Supplement: Supplemental Material [file KCBT_A_2061279_SM6877.zip › Supplemental_Figure_1.jpg]

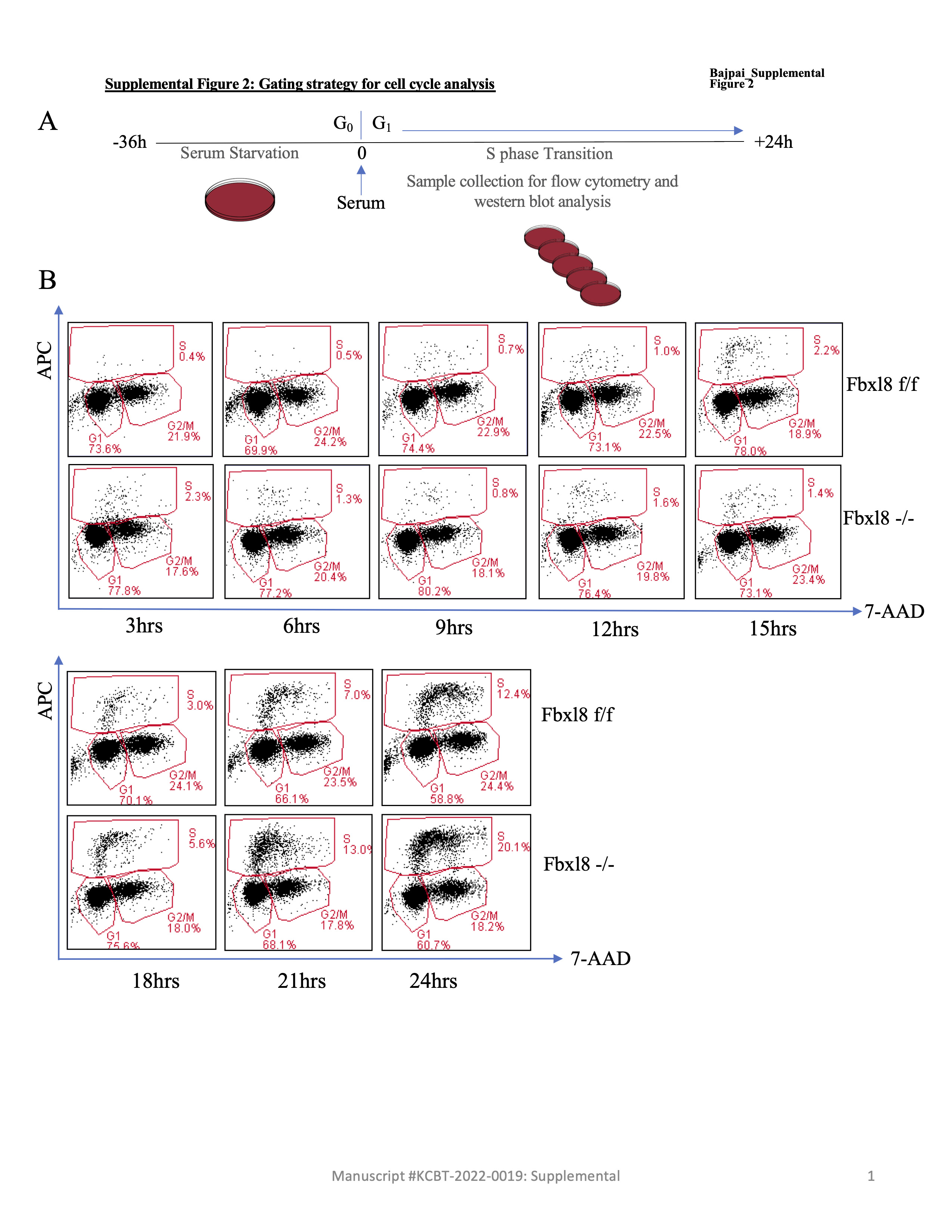

Supplement: Supplemental Material [file KCBT_A_2061279_SM6877.zip › Supplemental_Figure_2_300_dpi.jpg]

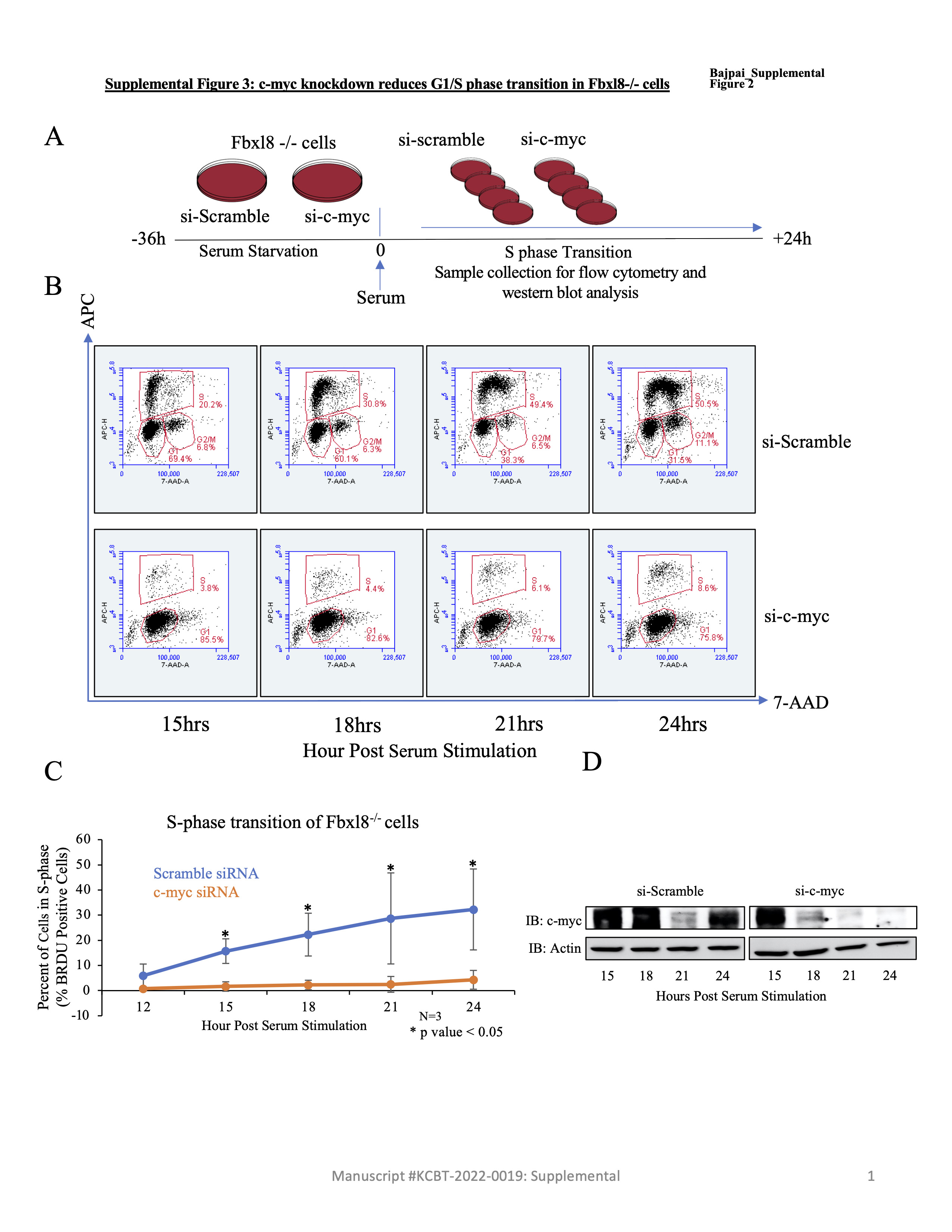

Supplement: Supplemental Material [file KCBT_A_2061279_SM6877.zip › Supplemental_Figure_3.jpg]
